# Caco-2 Invasion by *Cronobacter sakazakii* and *Salmonella enterica* Exposed to Drying and Heat Treatments in Dried State in Milk Powder

**DOI:** 10.3389/fmicb.2017.01893

**Published:** 2017-09-27

**Authors:** Emilie Lang, Stéphane Guyot, Pablo Alvarez-Martin, Jean-Marie Perrier-Cornet, Patrick Gervais

**Affiliations:** ^1^UMR PAM A 02.102 Procédés Alimentaires et Microbiologiques, Université de Bourgogne Franche-Comté/AgroSup Dijon, Dijon, France; ^2^Novolyze, Dijon, France

**Keywords:** *Salmonella enterica*, *Cronobacter sakazakii*, Caco-2, invasion, stress

## Abstract

Due to the ability of foodborne pathogens to survive in low moisture food, the decontamination of milk powder is an important issue in food protection. The safety of food products is, however, not always insured and the different steps in the processing of food involve physiological and metabolic changes in bacteria. Among these changes, virulence properties may also be affected. In this study, the effect of drying and successive thermal treatments on the invasion capacity of *Salmonella* Typhimurium, *Salmonella* Senftenberg, and *Cronobacter sakazakii* was assessed. Bacteria were dried on milk powder at three different water activity levels (0.25, 0.58, and 0.80) and heated at two different temperatures (90°C and 100°C) for 30 and 120 s. After recovery, stressed bacterial populations were placed in contact with Caco-2 cells to estimate their invasion capacity. Our results show that drying increases the invasion capacity of foodborne pathogens, but that heat treatment in the dried state did not exert a selective pressure on bacterial cells depending on their invasion capacity after drying. Taken together, our findings add to the sum of knowledge on food safety in dried food products and provide insight into the effects of food processing.

## Introduction

*Salmonella enterica* is a Gram-negative, facultative anaerobic, motile and non-spore forming bacteria which causes human salmonellosis. It is a major pathogen in the food industry and is highly represented in outbreaks across the world, with nearly 100,000 cases every year in the European Union alone (Beuchat et al., 2013). Its target population is principally composed of infants and young children (0 – 4 years old). Salmonellosis generally causes nausea, vomiting, abdominal cramps, diarrhea (sometimes necrotizing), fever and headache (Hohmann, 2001). Due to the low infective dose (1–10 cells) required to cause illness in infants and immunocompromised populations, *Salmonella* is an important issue for food safety (Akhtar et al., 2014). In addition, *Cronobacter* (formerly *Enterobacter sakazakii*), another Gram-negative, facultative anaerobic, motile and non-spore forming bacteria, is considered an opportunistic pathogen which can cause severe infections entailing a death rate of up to 80%, including meningitis, bacteremia or necrotizing enterocolitis in infants (Holý and Forsythe, 2014). The infective dose is unfortunately not well defined, and the incidence of this bacterium is largely underestimated. *Cronobacter* is also a major issue for producers of infant formula. These two bacteria are potential causes of severe infection following consumption of food products, especially powdered infant formula.

This is the reason why the *Codex Alimentarius*, which regulates food standards, imposes the absence of *Cronobacter* and *Salmonella* contamination in powdered infant formula, formula for special medical purposes and human milk fortifiers (Codex Alimentarius, 2008). Nevertheless, such contamination may elude food safety analysis (Cahill et al., 2008; Forsythe, 2014); in recent years, a certain number of cases of contamination by these two pathogens have been identified in infant formula and milk powder (a_w_ ≈ 0.25–0.45). Contaminations in milk may occur during the transfer to spray-drying, during the spray-drying and during dried milk handling. This is reflected in outbreaks involving *Cronobacter*, such as the outbreaks in 1986 in Iceland (3 cases), in 1988 in the United States (4 cases), in 1998 in Belgium (12 cases), in 2001 in the United States (11 cases), in 2004 in France (3 cases) or in 2008 in the United States (2 cases). Outbreaks of *Salmonella enterica* have also been reported, such as the 1976 outbreak in Trinidad (3,000 cases), that in 1986 in the United Kingdom (76 cases), in 2005 in France (141 cases) or in 2008 in Spain (42 cases), all due to PIF or milk powder that from part of low water activity food products (Podolak et al., 2010; Beuchat et al., 2013; Forsythe, 2014). *S. enterica* is also clearly linked to outbreaks involving other low moisture food products (Beuchat et al., 2013; Burgess et al., 2016).

Foodborne bacteria encounter many stresses in food processing environments and in food products (Humphrey, 2004). Drying is one such stress and takes place during low moisture food production and during environmental contamination. Drying consists in a diminution of environmental water activity (a_w_) which represents the water available for chemical and biochemical reactions. In a dried state, bacteria are more resistant to widely used decontamination processes, such as heat treatments (Rychlik and Barrow, 2005; Shaker et al., 2008; Guo and Gross, 2014). This resistance is partly due to the induction of a stress response by activation of the metabolic pathways which modify membrane composition and/or protein productions (Shen and Fang, 2012).

Stress perception also plays a role in other metabolic pathways, such as the activation of certain virulence genes governed by several two-component systems which sense environmental perturbations (Spector and Kenyon, 2012). For example, PhoQ-PhoP senses acid stress which is known to increase virulence properties in *Salmonella enterica*. In addition, EnvZ-OmpR, implied in osmotic change sensing, may control RNAm and protein regulating HilA, a central transcriptional activator in *S. enterica* virulence (Rychlik and Barrow, 2005). Ye et al. (2015) likewise suggest that osmotic changes are also related to the virulence of *C. sakazakii* (Ye et al., 2015). These authors directly observed that a virulence strain of this bacteria presented a higher expression and presence of EnvZ-OmpR than an attenuated strain (Giri et al., 2012).

In short, food processes can be stressful for foodborne pathogens and may impact bacterial virulence (Buchanan et al., 2000). Moreover, once in the dried state, a decontamination treatment is often applied to a dried food product to ensure food safety. As drying increases resistance to further decontamination treatment, it is possible to consider that the increase in virulence may impact pathogen survival of the heat treatment.

In this study, we describe the impact of drying and successive heat treatments on one virulence property of *C. sakazakii*, *S. enterica* subsp. *enterica* serovar Typhimurium and serovar Senftenberg. All experiments were performed in a food product dried at three different a_W_ levels (0.25, 0.58, and 0.80) and heated in the dried state at two different temperatures (90°C and 100°C). Invasion capacity in Caco-2 cells was subsequently performed using survival cells.

## Materials and Methods

### Strain Cultivations

*Salmonella enterica* subspecies *enterica* serovar Typhimurium DT104 DSM 10506, *Salmonella enterica* subspecies *enterica* serovar Senftenberg 775W DSM 10062 and *Cronobacter sakazakii* CIP 103183T strains were used in the present study. Two serovars of *S. enterica* were tested regarding their respective behavior toward drying and heat treatment showed in literature (Beuchat et al., 2013). *S*. Typhimurium was chosen for its high thermal resistance in dried state and its relevance in outbreaks linked to low-moisture foods. *S*. Senftenberg was chosen for its high thermal resistance (Ng et al., 1969). Finally, *C. sakazakii* was chosen for its resistance to stress and its relevance in outbreaks (Dancer et al., 2009). All cultures were stored in Tryptic Soy Broth (TSB, Sigma–Aldrich) with 20% glycerol (Sigma–Aldrich) at -80°C. For recovery, bacteria were inoculated on Tryptic Soya Agar (TSA, Sigma–Aldrich) at 37°C for 24 h; five colonies of each bacterium were subsequently picked up in 50 mL of Tryptic Soya Broth (TSB, Sigma–Aldrich) and incubated for 8 h at 37°C. Suspensions were then diluted in 50 mL of new TSB in order to reach an Optical Density (OD) of 0.01 at 600 nm. Cultures in the stationary phase were obtained after 20 h at 37°C.

### Inoculation of Powder

Milk powder was used in this study as a simplified model of dried food product. To obtain an inoculated milk powder (26% fat, Regilait, Saint-Martin-Belle-Roche, France), 50 mL cultures were centrifuged (3,400 *g*, 10 min at 25°C) and washed twice with 25 mL of PBS. In a final step, the supernatant was removed and cell pellets were weighed. Milk powder was added to the pellets at a 1:20 ratio (w_pellet_:w_powder_) and homogenized by means of a mortar for 30 min. Directly after inoculation, milk powder a_W_ was checked using an a_W_ meter (Aqualab, Decagon Devices, Inc, Dardilly, France) and found to be approximately 0.80. The cultivability of the bacteria was estimated using the spread plating method after incubation in TSA media for 24 h at 37°C and recorded as CFU/mL.

### Drying Process

To dry inoculated milk powder, hermetic boxes with saturated salt solutions at the bottom which regulated the a_W_ and consequently the atmosphere RH were used. Potassium acetate and sodium bromide (both from Sigma–Aldrich) were used in this study to reach an *a_w_* of 0.25 and 0.58. Atmospheres were maintained under convection using a ventilator (Sunon, Radiospare, France) as described in a previous study (Lemetais et al., 2012). For each strain, approximately 2 g of inoculated milk powder were spread on four small Petri dishes, which were then placed without the lids inside hermetic boxes for 16 h in order to reach the final a_W_ level. All drying processes were performed at room temperature.

### Thermal Treatment

0.1 g of dried inoculated milk powder (Régilait, France) were put into a 0.2 mL tube and treated at two different holding temperatures (90°C and 100°C) for 0, 30, and 120 s in a thermocycler (Bioer, France). Samples were then cooled to 4°C to stop the impact of the thermal treatment. Milk powder was rehydrated by adding 1 mL of PBS and agitating for 30 s, as recommended by the supplier.

### Virulence Assays

#### Caco-2 Cell Maintenance and Preparation

Caco-2 ACC 169 (DSMZ, Germany) was used in this study. Cells were maintained in flasks of 175 cm^2^ in a complete medium, comprised of Dulbecco’s Modified Eagle’s Medium (DMEM, Invitrogen, France) supplemented with 10% Fetal Bovine Serum (FBS, Invitrogen, France), 1% Minimal Essential Medium with Non-Essential Amino Acids (MEM NEAA 100X, Invitrogen, France) and 2 mM L-glutamine (Invitrogen, France). Flasks were maintained in a humidified atmosphere containing 5% CO_2_ at 37°C. At confluence, complete medium was removed and cells were washed three times in PBS (pH 7.2, Invitrogen, France). 5 mL of 0.25% Trypsin-phenol red (Invitrogen, France) were added to cover the cell layers and flasks were incubated 15 min in a humidified atmosphere containing 5% CO_2_ at 37°C, permitting to retrieve Caco-2 cells. The action of trypsin was stopped by adding 20 mL of complete medium. Viable cell concentration was estimated by means of trypan blue (Invitrogen, France). Caco-2 cells were seeded at a density of approximately 300,000 cells per well in 6-well tissue culture plates (Nunc, France), containing 3 mL of complete medium per well. Plates were incubated in humidified atmosphere containing 5% CO_2_ at 37°C for 15–17 days to obtain fully differentiated cell layers. During this period, the medium was changed every 2 days. Before use for virulence assays, cell layers were washed three times in PBS (pH 7.2) and 3 mL of complete medium without gentamicin were added.

#### Bacterial Sample Preparation

Control, dried and heated bacterial samples were washed three time in PBS by centrifugation (3,200 *g*, 10 min, 25°C). If necessary, several samples (taken from the same inoculated milk powder) treated in the same conditions were pooled together to reach the fixed bacterial concentration for the experiment. Counting by CFU was performed after incubation in TSA medium for 24 h at 37°C and results were expressed as log_10_(N_1_/N_0_), where N_1_ represented the CFU after drying and N_0_ represented the initial contamination of inoculated milk powder before drying. After thermal treatment, results were expressed as log_10_(N_2_/N_1_), where N_2_ represented the contamination of milk powder after heat treatment. For each virulence assay, the bacterial concentration of each sample was adjusted to 10^7^ CFU/mL. If necessary, several samples were pooled to reach this concentration.

#### Invasion in Caco-2 Cells

Ten microliter of prepared bacterial sample were added per well. Following incubation in humidified atmosphere containing 5% CO_2_ at 37°C for 1 h 30, 30 μL of 10 mg/L gentamicin (concentration per well of 100 μg/mL) were added before incubation in humidified atmosphere containing 5% CO_2_ at 37°C for 30 min. Medium was then removed and each well was washed three times with PBS (pH 7.2). Caco-2 cells were lysed with 1% Triton X-100 for 5 min. The cell lysates were then diluted and plated in TSA Petri dishes and incubated at 37°C for 24 h, permitting an estimation of the CFU.

### Statistical Analyses

All experiments were performed independently five times. The effects of the factors on the thermal treatment were evaluated for each bacterium by analysis of variance (ANOVA) using R v3.4.0 software (R Development Core Team, 2008). Significance was evaluated when the *p*-value was equal to or less than 0.05; in this case a Tukey’s HSD (Honest Significant Difference) test was performed to observe significant differences among conditions.

## Results

The effects of drying and heating in the dried state on the invasion of *C. sakazakii*, *S*. Typhimurium, and *S*. Senftenberg in Caco-2 cells are presented below.

### Impact of Drying on Cultivability and Bacterial Virulence

**FIGURE 1:**
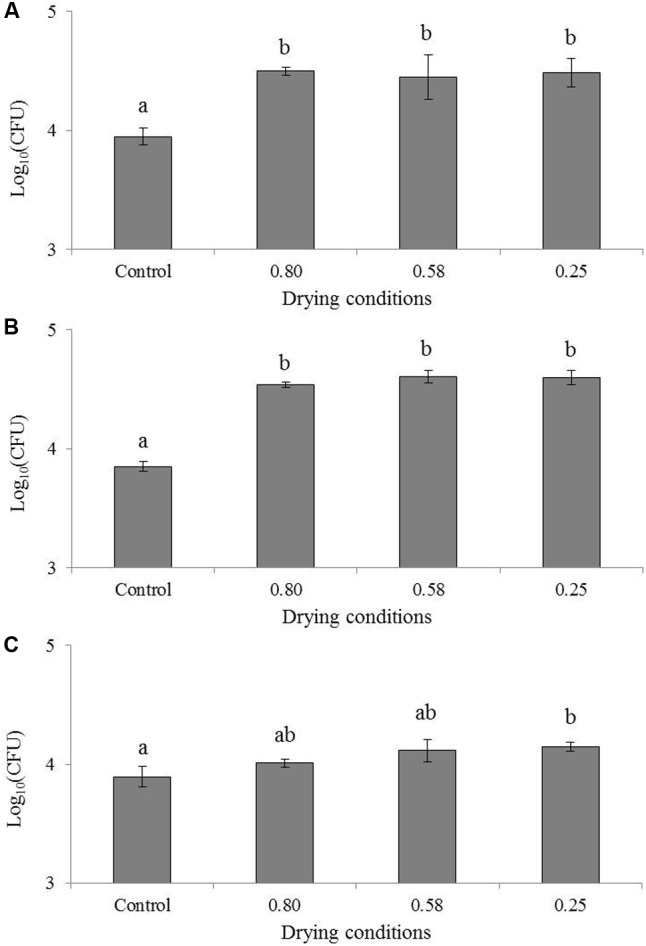
Impact of drying on the invasion of *Cronobacter sakazakii*, *Salmonella* Typhimurium and *Salmonella* Senftenberg in Caco-2 cells. Results of invasion after 1 h 30 are presented in log_10_(CFU) for the same inoculum of **(A)** *Cronobacter sakazakii*, **(B)** *Salmonella* Typhimurium and **(C)** *Salmonella* Senftenberg. “Control” represents the invasion of pure culture, “0.80” is invasion directly after the inoculation of milk powder, “0.58” is invasion directly after drying to an a_W_ of 0.58 and “0.25” is invasion directly after drying to an a_W_ of 0.25. Error bars represent the standard deviations calculated on independent triplicates. Letters above bars represent the significant differences among conditions resulting from a Tukey’s HSD test.

The effect of drying on the invasion capacity of the studied foodborne pathogens is presented in **Figure 1**. ANOVA tests revealed a significant effect of the water activity level of the decimal logarithm of the bacterial population found after the invasion test. In the case of *C. sakazakii* (**Figure 1A**), the HSD test revealed two significantly different groups. The first group is represented by the pure culture, with a count of 3.9 log bacteria (“Control” in **Figure 1A**) after the invasion test, and the second is composed of the three different a_W_ levels (i.e., 0.80, 0.58, and 0.25) with a mean count of approximately 4.4 log bacteria. The difference between the means of the two HSD groups represented a count difference of 0.52 log. In the case of *S.* Typhimurium (**Figure 1B**), the HSD test revealed two significantly different groups as well. The first group is the pure culture, with 3.8 log bacteria (“Control” in **Figure 1B**) after the invasion test. The second group is composed of the three different a_W_ levels with a count mean of approximately 4.3 log bacteria. In this case, the difference between the means of the two HSD groups represented a count difference of approximately 0.54 log. For *S.* Senftenberg (**Figure 1C**), the HSD test revealed two significantly different groups. The first one is composed of the pure culture and drying at 0.80 a_W_, with counts of 3.9 and 4.0 log bacteria respectively after the invasion test (“Control” in **Figure 1C**). The second group is composed of the three different drying levels with a mean count of approximately 4.2 log bacteria. The difference between the means of the two HSD groups represented approximately 0.20 log. No differences were observed among the different drying levels, and the drying level at a_W_ = 0.80 belonged to both groups. This signifies that drying significantly increased invasion capacity at the beginning of the drying process in the three tested bacteria. This increase in invasion capacity was induced from the beginning of the drying as the effect was already significant for drying to an a_W_ of 0.80 in *C. sakazakii* and *S*. Typhimurium and between 0.80 and 0.58 in *S*. Senftenberg. Under this a_W_ level (0.25 and 0.58), no extended effect of drying was observed.

**FIGURE 2:**
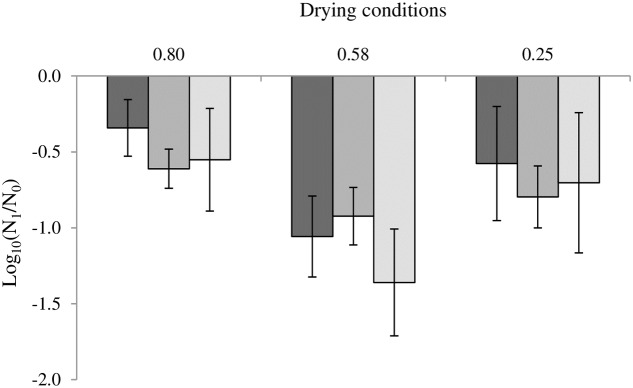
Impact of drying on the cultivability of *Cronobacter sakazakii*, *Salmonella* Typhimurium and *Salmonella* Senftenberg. From darker to lighter, the results are presented for *Cronobacter sakazakii*, *Salmonella* Typhimurium and *Salmonella* Senftenberg. Error bars represent the standard deviations calculated on independent triplicates.

The loss of cultivability after drying at 0.25, 0.58 and 0.80 a_W_ is presented in **Figure 2**. In *C. sakazakii*, the loss of cultivability represented -0.57 log, -1.06, and -0.34 log after drying at 0.25, 0.58, and 0.80 a_W_, respectively, while the virulence increase was represented by an invasion increase of 0.52 log in both cases (**Figure 1A**). In the same way, in the case of *S*. Typhimurium, at water activity levels of 0.25, 0.58, and 0.80, the uncultivable bacteria represented -0.83 log, -1.02 and -0.61 log of the initial bacteria respectively, while the virulence increase represented 0.54 log of invasive bacteria (**Figure 1B**). In *S*. Senftenberg, the invasion increase compensated for the loss of cultivability, which measured approximately -0.70 log, -1.38 and -0.55 log after drying at 0.25 and 0.58 a_W_, respectively (**Figure 1C**).

### Impact of Heating in the Dried State on the Cultivability and Virulence of Bacteria

**FIGURE 3:**
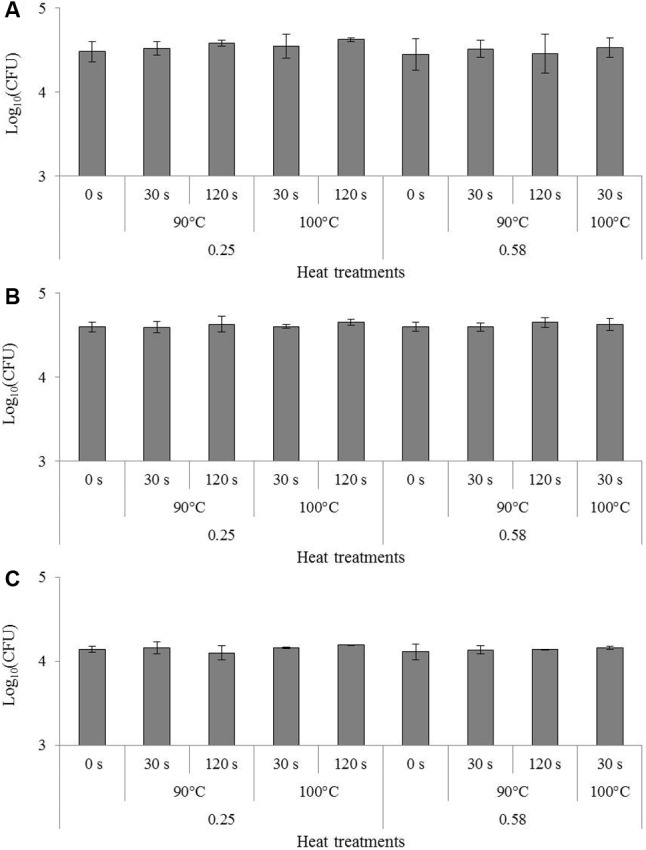
Impact of heating in the dried state on the invasion of *Cronobacter sakazakii*, *Salmonella* Typhimurium and *Salmonella* Senftenberg in Caco-2 cells. Results of invasion after 1 h 30 are presented in log_10_(CFU) for the same inoculum of **(A)** *Cronobacter sakazakii*, **(B)** *Salmonella* Typhimurium and **(C)** *Salmonella* Senftenberg. Error bars represent the standard deviations calculated on independent triplicates. Letters above bars represent the significant differences among conditions resulting from a Tukey’s HSD test.

The effect of heating in the dried state on the invasion capacity of studied foodborne pathogens is presented in **Figure 3**. ANOVA tests revealed no significant effect of heating conditions compared to previous drying (represented by “0 s” in **Figure 3**) on any of the bacteria after the invasion test. More exactly, heating did not increase the invasion capacity of dried bacteria.

**FIGURE 4:**
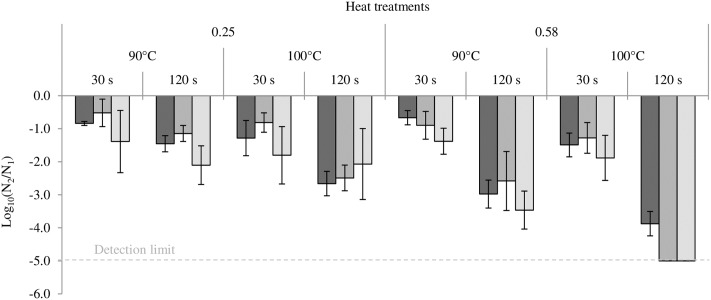
Impact of heating on the cultivability of *Cronobacter sakazakii*, *Salmonella* Typhimurium and *Salmonella* Senftenberg. From darker to lighter, the results are presented for *Cronobacter sakazakii*, *Salmonella* Typhimurium and *Salmonella* Senftenberg. Error bars represent the standard deviations calculated on independent triplicates.

The impact of heat treatment on bacterial cultivability is presented in **Figure 4**. The loss of cultivability was greatest in all bacteria at an a_W_ of 0.58. In the same way, this loss was greatest at high temperature (100°C) and for long treatment time (120 s). In all bacteria, the least loss of cultivability was observed in milk powder at an a_W_ of 0.25 treated at 90°C for 30 s. After 120 s at 100°C in milk powder at 0.58, cultivability of both *Salmonella* serovars was under the detection limit of the method. The loss of cultivability in all bacteria was greater than 1 log decrease and this loss of cultivability was in no case compensated for by an increase in virulence.

## Discussion

### Drying and Invasion Capacity of Foodborne Pathogens

A threshold effect of a_W_ decrease was detected, higher than 0.80 for *S*. Typhimurium and *C. sakazakii*, and between 0.80 and 0.58 for *S*. Senftenberg and could correspond to the water activity limit above which metabolic pathways are still active, involving certain enzymes which require a lower water activity threshold to function (Lee and Kim, 1995).

Foodborne pathogens must adapt to several and successive environmental stresses in foods, gastrointestinal tract, and also host’s phagosome to cause illness. All these stresses engage different and coordinate gene activation and expression pathways, as for acid, osmotic, antimicrobial peptide, or also nutrient starvation stresses. These perturbations activate generally several alternative metabolic pathways involved in stress adaptation, promoting activation or repression of multiple genes in response to a single environmental perturbation (Rychlik and Barrow, 2005; Shen and Fang, 2012; Guo and Gross, 2014; Feeney et al., 2015; Burgess et al., 2016). Regarding *S. enterica*, desiccation induces up-regulation of gene and transcriptional factors entailed in other stress responses, *rpoH* and *rpoE* genes encoding transcriptional factors involved in heat and oxidative stress response, respectively (Gruzdev et al., 2012). Also, Aviles et al. (2013) demonstrated that storage of *Salmonella* biofilm at a_W_ = 0.3 promoted an increased expression of stress response genes *rpoS* and *otsB* correlated with survival, indicating cross-protection to desiccation and acid stress. Also, in *Cronobacter*, a cross-protection is observed for several stresses. For example, Alvarez-Ordóñez et al. (2014) showed that *C. sakazakii* exposed to acid stress produces activation of several genes encoding chaperone proteins, as DnaJ also involved in heat stress response. Consequently, the way that bacteria undergo environmental perturbation as performed in this study could also promote their survival in gastrointestinal tract until epithelium cells.

Many stresses are known to impact the virulence capacity of foodborne pathogens, especially in the case *S. enterica* which is well studied (Rychlik and Barrow, 2005). The most completely studied stress response is acid stress response, occurring during the passage in the gastrointestinal track and which is under the control of the general stress response transcriptional factor RpoS (Fang et al., 1992; Gahan and Hill, 1999; Koutsoumanis and Sofos, 2004; Rychlik and Barrow, 2005). Moreover, RpoS is already shown to be linked with the invasion properties of bacterial cells and also known to play a role in osmotic stress, by activating the accumulation of ions and compatible solutes through *proU* activation (Csonka, 1989; Rychlik and Barrow, 2005; Du et al., 2011; Shen and Fang, 2012; Andino and Hanning, 2015). Consequently, osmotic stress could also activate such virulence properties. Although several studies have examined the impact of osmotic stress in liquid media (i.e., water/glycerol or salt solution), few are especially focused on desiccation. It seems that during desiccation, activation of the RpoE regulon is also recorded and this could impact the virulence property of *S. enterica* for which the RpoE transcriptional factor is essential for success inside the host (Li et al., 2015). Regarding *C. sakazakii*, the ability of this bacterium to survive osmotic or desiccation stress is often considered as remarkable compared to other Enterobacteriaceae (Burgess et al., 2016). *C. sakazakii* responds similarly to *Escherichia coli* or *Salmonella enterica*, i.e., first accumulating potassium and counter-ion, and secondly accumulating/synthesizing compatible solutes (Feeney et al., 2014). Nevertheless, the regulation pathways are not completely elucidated in *C. sakazakii*. It was already shown that *hfq* gene plays an important role in virulence and stress acclimation. Indeed, Kim et al. (2015) have showed that Δ*hfq* mutant presented a three-fold attenuation of invasion in animal cells and a lower resistance to oxidative stress (hydrogen peroxide, 100-fold). They also suggest that this gene plays an important role in regulation of multiple genes participating in virulence (Kim et al., 2015).

Thus, the way to undergo and survive drying could interfere in bacterial virulence. Indeed, Beaubrun et al. (2017) did not detect differences at the genomic level between two *Salmonella* Montevideo species (SAL242S and SAL242, atypical mucoid and non-mucoid strains, respectively). Nevertheless, they detected transcriptomic difference between these species, notably increased expression of EPS and SPI1 genes by SAL242S, responsive to mucoid and virulence protein production. They hypothesized that this is associated with post-transcriptional factors induced during environmental stress and that this increased expression of SPI1 genes may play a role in protecting *Salmonella* from environmental stressors (Beaubrun et al., 2017). Moreover, in studies concerning the transcriptome of desiccation stress, it has been clearly demonstrated that virulence is related to the stress response pathway (Li et al., 2012; Aviles et al., 2013). In *Cronobacter*, little knowledge was available. A recent study of Jing et al. (2016) showed that interaction between *C. sakazakii* and human intestinal epithelial cells induces modification of the bacterium transcriptome. Among upregulated genes, genes involved in the osmotic stress acclimation were identified. For instance, *proV* was upregulated 12-fold. This gene encodes for a glycine betaine/proline transport system ATP-binding protein, where glycine betaine and proline correspond to compatible solutes accumulated during osmotic stress (Feeney et al., 2014). In the same way, *kdpA*, *kdpB*, and *kdpC* were upregulated 27-, 15-, and 8-fold, respectively. These genes are associated with potassium transport system, implicated in first response to osmotic stress (Csonka and Hanson, 1991). Finally, Jing et al. (2016) also demonstrated that *betB* involved in the biosynthesis of osmoprotectant glycine betaine (Lamark et al., 1991) was upregulated 7-fold. Betaine aldehyde dehydrogenase (BetB) is an efficient osmotic regulator, which participates in catalyzing the oxidation of betaine aldehyde to glycine betaine (Kempf and Bremer, 1998). Taken together, all these informations suggest that genes associated with osmotic stress are important to infection of human intestinal epithelial cells by *C. sakazakii*. Consequently, resistance to drying could be linked to the disease-causing potential of the bacteria *C. sakazakii* and *S*. *enterica* and *vice versa*.

It is necessary to step back that drying has a potential negative impact on food safety and that drying conditions have to be managed to insure a minimal activation of several metabolic pathways which may be involved in increased invasion capacity. In addition, in our drying conditions the first step of a_W_ decrease was slow, i.e., 30 min to reach an a_W_ of 0.80. It is possible to assume that, as in the case of cultivability (Lang et al., 2017), a rapid drying of food products to reach a water activity under a physiologic threshold could limit the acclimation and the invasion capacity increase of foodborne pathogens.

Finally, rehydration of milk powder plays an important role for bacterial survival. Indeed, it was already shown that kinetics of rehydration can play on bacterial survival. The slower kinetics, the greater inactivation (Lang et al., 2016; Zoz et al., 2016). Previous works have also demonstrated that the temperature of rehydration allows an optimal bacterial survival in the approximate range of 35–45°C, depending on the bacterial strains.

### Heat in Dried State and Invasion Capacity of Foodborne Pathogens

Even if drying leads to a microbial stability of food products over time, this process does not sterilize the product. Consequently, low moisture foods are microbiologically stable, but not microbiologically safe (Beuchat et al., 2013). For this reason, these food products, and particularly herbs and spices, are often exposed to a subsequent decontamination process (as heat treatment) (Grasso et al., 2014; Niemira, 2014). Indeed, dried food products are heated to insure food safety. Our results showed that heating did not increase the invasion capacity of dried bacteria, which can be explained by the low water activity aborting physiological metabolism; nor did heating decrease invasion capacity, which may be explained by a protection by drying of structures involved in the virulence pathway. Microorganisms are more resistant to the decontamination process when water activity is low. This is partially due to the low water activity which stabilizes physiological structures and also due to cross protection mechanisms. Notably, osmotic stress encourages the synthesis of HSP (Heat Shock Proteins), a response which protects proteins and membrane from heat alteration (Richter et al., 2010; Shen and Fang, 2012). The fact that bacteria are able to engage certain mechanisms during drying also suggests that synthesized proteins may positively or negatively impact heat resistance. Indeed, virulence mechanisms involve the synthesis of several proteins and factors which could modify the membrane or cytosol composition and consequently influence stress resistance. For example, as some virulence proteins are membrane proteins, a modification of the membrane composition could impact the membrane phospholipid transition phase (Ibarra and Steele-Mortimer, 2009; Fàbrega and Vila, 2013) and, subsequently, impact bacterial resistance. In our case, no difference was detected between heated bacteria and dried bacteria, suggesting that the activation of virulence during drying does not interfere with heat sensitivity. Consequently, thermal treatment guarantees a great loss of cultivability and also does not increase the invasion properties of these two foodborne pathogens. If a maximal loss of cultivability can be guaranteed in dried food, thermal treatment could represent an effective tool in the quest for food safety.

## Conclusion

This study has analyzed the impact of drying and successive heat treatment in three foodborne milk powder pathogens. While the impact of several stresses on virulence has already been demonstrated, this is the first record of the impact of aerial drying and of heating in the dried state on the invasion capacity of *Salmonella enterica* and *Cronobacter sakazakii*. Our results show that drying to the 0.80 water activity level significantly increases the invasion capacity of these bacteria (approximately four times as many bacteria entering Caco-2 cells), which almost compensates for the loss of cultivability observed after drying. Further deeper drying and subsequent heat treatments did not modify the invasion capacity of the three studied bacteria and did not offset the loss of cultivability noted after heat treatment. Taken together, these results provide a new perspective on food processing as well as insight into its impact on health in terms of foodborne pathogens in dried food products. Further experiment in other dried food products, such as PIF, spices, herbs, or flour, will permit to strengthen this study and our knowledge regarding bacterial virulence, food transformation, food conservation, and link with food safety.

## Author Contributions

Substantial contributions to the conception or design of the work: EL and PG; the acquisition: EL; analysis and interpretation of data for the work: EL, SG, PA-M, J-MP-C, and PG. Drafting the work or revising it critically for important intellectual content: EL, SG, PA-M, J-MP-C, and PG. Final approval of the version to be published: EL, SG, PA-M, J-MP-C, and PG. Agreement to be accountable for all aspects of the work in ensuring that questions related to the accuracy or integrity of any part of the work are appropriately investigated and resolved: EL, SG, PA-M, J-MP-C, and PG.

## Conflict of Interest Statement

The authors declare that the research was conducted in the absence of any commercial or financial relationships that could be construed as a potential conflict of interest.
